# Feasibility of the Understanding and Managing Adult ADHD Programme: open-access online group psychoeducation and acceptance and commitment therapy for adults with attention-deficit hyperactivity disorder

**DOI:** 10.1192/bjo.2024.743

**Published:** 2024-09-26

**Authors:** Christina Seery, Aisling Leonard-Curtin, Lauren Naismith, Nora King, Fiona O'Donnell, Brendan Byrne, Christine Boyd, Ken Kilbride, Margo Wrigley, Louise McHugh, Jessica Bramham

**Affiliations:** UCD School of Psychology, University College Dublin, Ireland; ADHD Ireland, Dublin, Ireland; HSE National Clinical Programme for ADHD in Adults, Health Service Executive, Dublin, Ireland

**Keywords:** Attention-deficit hyperactivity disorder, adult ADHD, acceptance and commitment therapy, psychoeducation

## Abstract

**Background:**

Psychoeducational interventions are a critical aspect of supporting adults with attention-deficit hyperactivity disorder (ADHD). The Understanding and Managing Adult ADHD Programme (UMAAP) is a six-session, group-based webinar intervention that incorporates psychoeducation with acceptance and commitment therapy. UMAAP relies on self-referrals and is facilitated by a charity, to promote accessibility.

**Aims:**

The present study aimed to evaluate the feasibility of UMAAP and explore preliminary effectiveness.

**Method:**

Adults with formally diagnosed or self-identified ADHD (*n* = 257) participated in an uncontrolled pre–post design. Feasibility was indicated by attendance, confidence in completing the home practice and satisfaction. Quality of life, psychological flexibility, self-acceptance and knowledge of ADHD were assessed at baseline, 1 week post-intervention and 3 months later, to explore preliminary effectiveness.

**Results:**

Feasibility was demonstrated by the high attendance ratings and satisfaction with the intervention, although there was only moderate confidence in the ability to complete the home practices. Quality of life (mean increase 9.69, 95% CI 7.57–11.80), self-acceptance (mean increase 0.19, 95% CI 0.10–0.28) and knowledge of ADHD (mean increase 1.55, 95% CI 1.23–1.82) were significantly improved post-intervention. The effects were maintained at the 3-month follow-up. Psychological flexibility did not significantly change immediately post-intervention, but increased significantly at the 3-month follow-up (mean increase 0.42, 95% CI 0.26–0.58).

**Conclusions:**

Overall, UMAAP is a feasible intervention for adults with ADHD. Findings highlighted the feasibility of delivering psychological interventions online in group settings, to increase access to support for adults with ADHD.

Attention-deficit hyperactivity disorder (ADHD) is a form of neurodivergence associated with differences in attention regulation, hyperactivity and impulsivity.^1^ Adults with ADHD have an increased risk of experiencing significant mental health difficulties, such as anxiety, depression and substance misuse.^2^ Psychoeducation is recommended as a first step in care for adults diagnosed with ADHD.^3^ As such, psychological interventions with psychoeducational components are vital in supporting the well-being of adults with ADHD. Several studies have shown the effectiveness of psychological interventions for adults with ADHD. For example, cognitive–behavioural therapy (CBT) has demonstrated improvements in participants’ levels of anxiety and depression.^4^ Third-wave CBTs, like dialectical behavioural therapy (DBT) and acceptance and commitment therapy (ACT), are similarly effective in supporting adults with ADHD.^5–7^ However, psychological interventions have primarily been conducted in outpatient psychiatric clinics.^6,8,9^ Given the increasing demand on specialist services for ADHD, there may be value in developing and evaluating an accessible community-based psychological intervention for adults with ADHD.

## Increasing access to psychological interventions

With the rise in awareness of ADHD, more adults are seeking diagnoses and support for the challenges they experience, particularly marginalised groups who have been historically underdiagnosed.^10^ This has increased the pressures on busy services and further lengthened wait times,^11^ limiting the ability of adults with ADHD to access psychological support. Along with growing demand, geographical area can influence individuals’ ability to access adequate treatment for their ADHD.^12^ As such, open-access psychological interventions, where attendees self-refer to the programme, could help to reduce pressures on services. Online interventions have the advantage of being easily scalable and can overcome barriers such as lack of access to specialist care in a geographical area, and therefore can be open-access.

Online psychological interventions for adults with ADHD have been shown to improve difficulties with attention deficits compared with waitlist control groups.^13^ For example, in addition to improving symptoms, a self-guided intervention with components from CBT, DBT and goal management training improved levels of quality of life, and over half of the participants experienced reliable change.^14^ Additionally, group-based internet-delivered CBT (iCBT) has been shown to be as effective as individual iCBT for adults with ADHD.^15^ Group psychological inventions are cost-effective,^16^ and adults with ADHD have described the experience of meeting others with ADHD in interventions as very beneficial.^17^ Therefore, an open-access, group-based online intervention may have multiple benefits.

A feasibility study of a proposed online intervention for young adults with ADHD found that participants preferred that online interventions were guided by a clinician, rather than self-guided.^18^ A challenge of conducting a clinician-led, group-based online intervention is managing clinical risk. In an out-patient psychiatry setting, group facilitators can moderate the group and follow up with disclosures of risk. This is more challenging if the intervention is facilitated remotely and not linked to a particular service. As such, hosting the intervention as a webinar, in which the clinician leads psychological exercises and provides psychoeducation and attendees can interact via a moderated chat, could mitigate this risk while providing an accessible intervention.

## The Understanding and Managing Adult ADHD Programme

The Understanding and Managing Adult ADHD Programme (UMAAP) is a novel psychological intervention for adults with ADHD. UMAAP aims to provide accessible psychological support to adults with ADHD, and is hosted by a non-profit organisation in collaboration with public health services. The programme involves six weekly webinars, in which the attendees receive psychoeducation about ADHD and ACT principles. The psychoeducational content was derived from the priorities of adults with ADHD.^19^ Given UMAAP's novel nature, a mixed-methods research programme was conducted to evaluate its feasibility. Qualitative research was used to provide insight into participants’ views of therapeutic mechanisms, as well as barriers and facilitators to engagement.^20^ The quantitative element of the research programme sought to measure the various elements of feasibility and acceptability, such as attendance rates, satisfaction and preliminary effectiveness.^21^

The present study aimed to quantitatively explore the feasibility, acceptability and preliminary effectiveness of a novel, open-access, online, group-based psychological intervention. The first objective was to assess levels of attendance, participants’ ratings of helpfulness and overall satisfaction. The second objective was to explore preliminary evidence for effectiveness, with outcome measures of quality of life, psychological flexibility, self-acceptance and knowledge of ADHD.

## Method

The present study presented the quantitative findings of an acceptability and feasibility research programme evaluating UMAAP (OSF pre-registration: osf.io/c48b6). The research programme aimed to establish if UMAAP is feasible as an intervention for adults with ADHD, using an uncontrolled pilot study design. The authors assert that all procedures contributing to this work comply with the ethical standards of the relevant national and institutional committees on human experimentation and with the Helsinki Declaration of 1975, as revised in 2013. All procedures involving human patients were approved by the first and senior authors’ host institution human research ethics committee (HS-21-01-Seery-Bramham). This research was completed as part of the first author's PhD thesis.^22^ Participant inclusion criteria were as follows: registered to attend UMAAP, age over 18 years, residing in Ireland, fluent in English and has diagnosed or self-identified ADHD.

### Intervention

UMAAP is a 6-week, webinar-based psychological intervention. UMAAP was developed as a collaboration between University College Dublin, the HSE National Clinical Programme for ADHD in Adults and ADHD Ireland, to provide an evidence-based intervention available within a community setting. Attendees registered themselves for the programme and can be formally diagnosed or self-identify as having ADHD. UMAAP is hosted by ADHD Ireland. UMAAP was facilitated by either a senior psychologist or a certified mindfulness practitioner, both of whom have ADHD, using the teleconferencing platform Zoom. A pilot cohort in November 2021 recommended that UMAAP be facilitated by a psychologist or therapist with ADHD, or co-facilitated by a peer with ADHD. As such, qualified facilitators with ADHD were recruited. The facilitators shared personal examples of having ADHD during sessions, such as unwanted thoughts related to their ADHD or experiences of executive functioning differences, to reduce internalised stigma and promote self-acceptance. An assistant psychologist supported their facilitation by managing the chat function, breakout rooms and attendees’ inquiries between sessions.

The overall aim of UMAAP was to increase the quality of life in adults with ADHD by improving their knowledge and self-acceptance of adult ADHD. Each of the weekly sessions lasted for 1.5 h, with an optional 15-min question and answer session participants could stay for. The facilitator opened the session with a mindfulness practice, before providing participants with psychoeducation about adult ADHD and ACT principles, supported by PowerPoint slides. Exercises related to the psychoeducation were built into the sessions, and participants were given an accompanying guidebook with templates for completing the exercises. Attendees could comment on the content, share their experiences or ask questions in the teleconferencing chat box, which was monitored by the assistant psychologist who asked the facilitator the question or summarised key themes of the chat. Halfway through the session, participants opted into a brief breakout room with other attendees or chose to watch a video. Attendees were not permitted to turn on their microphones because of the size of cohorts. Attendees could also email the assistant psychologist between sessions with questions, which are discussed at the beginning of the next session. Table 1 provides a brief of the content covered within the intervention, previously published in the qualitative evaluation.^20^ A detailed description of session content is available in Supplementary File 1 available at https://doi.org/10.1192/bjo.2024.743.

**Table 1:** Overview of Understanding and Managing Adult ADHD Programme content

Session	Session content
1. Foundations of UMAAP	Executive functioning Workability of participants’ attitudes toward their ADHD Self-management styles
2. ADHD acceptance	Accepting adult ADHD ACT for ADHD Values-guided actions
3. Values and vulnerabilities	Values-guided actions Self-as-context Acceptance Defusion Committed action
4. Unwanted thoughts	Defusion Self-compassion
5. Unwanted emotions	Emotional regulation Acceptance Connectedness to the present moment
6. Conclusion	Review of programme content Committed action

UMAAP, Understanding and Managing Adult ADHD Programme; ADHD, attention-deficit hyperactivity disorder; ACT, acceptance and commitment therapy.

### Overview of UMAAP content

Ten cohorts, hosted from April 2022 to June 2023, were included in the research evaluation.^23^ The initial cohort had a capacity of 21 adults with ADHD, whereas cohorts 2–5 gradually increased capacity from 30 to 46 attendees, and cohorts 6–10 had a maximum of 50 registrants. The average group size was 42 attendees.

### Measures

#### Within-session evaluations

Attendance was noted for each session and compared with the number of people who attended the first session. To determine each session's acceptability, participants were asked to answer an anonymous poll, with questions based on in-session assessments of problem severity, confidence and helpfulness.^23^ At the beginning and end of each session, attendees were asked to rate on a scale of 1–5 how big of a challenge having ADHD feels like. At the end of the session, they were also asked if anything in the session helped with this feeling of challenge (yes or no), how confident they feel that they will complete the home practice (on a scale of 1–5) and how helpful was the session overall (on a scale of 1–5). Participants’ ratings of how helpful the session was were totalled across the programme, to indicate overall satisfaction. The highest score was 30. Like Smith et al,^24^ we subdivided the total score into four satisfaction categories: ‘poor’ (score 10–14), ‘fair’ (score 15–20), ‘good’ (score 20–24) and ‘excellent’ (score 25–30).

#### Adult ADHD Quality of Life Scale

The Adult ADHD Quality of Life Scale (AAQoL)^25^ is a 29-item questionnaire that aims to assess the quality of life in adults with ADHD across four domains of life productivity, psychological health, relationships and life outlook. Items are rated on a scale of not at all/never to extremely/very often. To establish subscale and overall scores, items are transformed into a 0–100 point scale, with higher scores indicating greater quality of life. The AAQoL's internal reliability for the present study's baseline measures was good (Cronbach's *α* = 0.85).

#### Psychological flexibility

The Psychological Flexibility scale (Psy-Flex)^26^ is a six-item questionnaire that aims to measure psychological flexibility in a contextually sensitive manner. Each item reflects a core ACT skill, and participants are asked to rate their experiences of the skill in the past 7 days on a scale of very often to very rarely. The Psy-Flex's internal reliability for the present study's baseline measures was acceptable (Cronbach's *α* = 0.78).

#### Disability Self-Acceptance Scale

The Disability Self-Acceptance Scale (DSES)^27^ is a 10-item questionnaire to measure self-acceptance in relation to disabilities. Participants indicate their level of acceptance on a scale of strongly agree to strongly disagree, with higher scores indicating greater self-acceptance. For the present research, the items’ language was changed from ‘my disability’ to ‘my ADHD’. The DSES's internal reliability for the present study's baseline measures was moderate, and therefore findings should be interpreted with caution (Cronbach's *α* = 0.69).

#### Knowledge of ADHD

Participants were asked to rate how much they feel they know about ADHD on a scale of one (nothing at all) to 10 (a great deal).

### Procedure

ADHD Ireland advertised UMAAP on their website and clinicians recommended UMAAP to individuals with diagnosed or suspected ADHD they felt may benefit. Adults with ADHD interested in participating in UMAAP contacted the assistant psychologist, who allocated them to a brief waiting list, after which they were registered to a rollout conducted at a time and day that suited them. Once registered to a rollout, the assistant psychologist invited all attendees to anonymously participate in the research evaluation. An *a priori* power analysis, using G*Power version 3.1 for Mac OS (Heinrich-Heine-Universität Düsseldorf, Germany; https://www.psychologie.hhu.de/arbeitsgruppen/allgemeine-psychologie-und-arbeitspsychologie/gpower.html) to determine the minimum sample size (criteria: *α* = 0.05, power: 0.95, effect size: 0.25), indicated that at least 43 participants were necessary for statistical power. Teresi et al recommend sample sizes of at least 70 people in feasibility studies evaluating process outcomes, such as acceptability and adherence.^28^ The research team initially aimed to recruit 100 participants to allow for drop-out, as reported in the pre-registration. There was significant demand for UMAAP once registration opened, and as such, the research team decided to recruit ten cohorts to allow for diversity in views and responses to UMAAP, particularly because of the large cohort size.

Participants provided written informed consent and completed the baseline survey by answering the questionnaires and demographic questions (time point 1). Participants were asked to consent to be contacted directly by the research team with an invitation to participate in the second-round data collection, which was conducted within a week of participants finishing UMAAP (time point 2). Participants who consented to be contacted provided their email addresses and created a unique identifier code to link their data across time. The post-UMAAP survey included the original measures in the baseline data collection and additional questions on participants’ qualitative experiences and their satisfaction with UMAAP. Individuals who participated in the post-UMAAP data collection were asked to consent to be contacted for a final round of data collection (time point 3). The final round was conducted 3 months after the participant had attended UMAAP, in which participants completed the questionnaires once more. All surveys were conducted online, using Qualtrics version October 2023, for Mac OS (Qualtrics, Utah, USA; https://www.qualtrics.com/).^29^ Five participants requested to complete the measures by telephone with the first author.

### Statistical analysis

SPSS version 28 for Mac OS^30^ was used to clean the data, conduct preliminary analysis, analyse missing data, and conduct multiple imputation and hypothesis testing. Descriptive statistics were used to analyse attendance rates, confidence in completing home practices, perceived helpfulness and satisfaction ratings. Independent *t*-tests and *χ*^2^-tests of independence were used to explore any differences between completers (participants who completed time points 2 or 3) and non-completers (participants who dropped out of the study after time point 1).

The data were assumed to be missing at random, and that missingness was associated with information contained within the data-set. The proportion of missing data by variable is reported in Supplementary File 2. As such, multiple imputation was adopted as the missing data treatment. Multiple imputation was used for variables missing less than 40% of data. ‘ADHD status’ was treated as an auxiliary variable. Predictive mean matching was used as a model for multiple imputation, with five imputations and ten iterations. As recommended by Woods et al,^31^ the descriptive statistics and main effects results with both complete cases and multiple imputations are reported in Supplementary File 2.

Repeated-measures analysis of variance (ANOVA) was conducted to test differences in outcome scores at all time points. Effect sizes were measured with partial eta-squared (*η*^2^_p_). Data visualisations were created with the ggplot2 package^32^ in RStudio, version 2023.12.0+369 for Mac OS (Posit, Vienna, Austria; https://dailies.rstudio.com/version/2023.12.0+369/).^33^

## Results

### Participants

In total, 257 adults with ADHD completed the baseline measures before initiating UMAAP. Ages ranged from 18 to 76 years (mean 41.16 years, s.d. = 10.07). The majority of participants identified as women (*n* = 187), with 57 men and five non-binary people. Most participants reported that they were formally diagnosed as having ADHD (*n* = 171) versus self-identifying as having ADHD but not being formally diagnosed (*n* = 81). Of those who were formally diagnosed and reported their ADHD type, 67% had combined type, 30% had inattentive type and 3% had hyperactive–impulsive type. Nearly half of participants (46%) identified as having an additional form of neurodivergence, such as autism or dyslexia. Participants reported attending a mean of five sessions (s.d. = 0.99, range: 1–6).

**Fig. 1:**
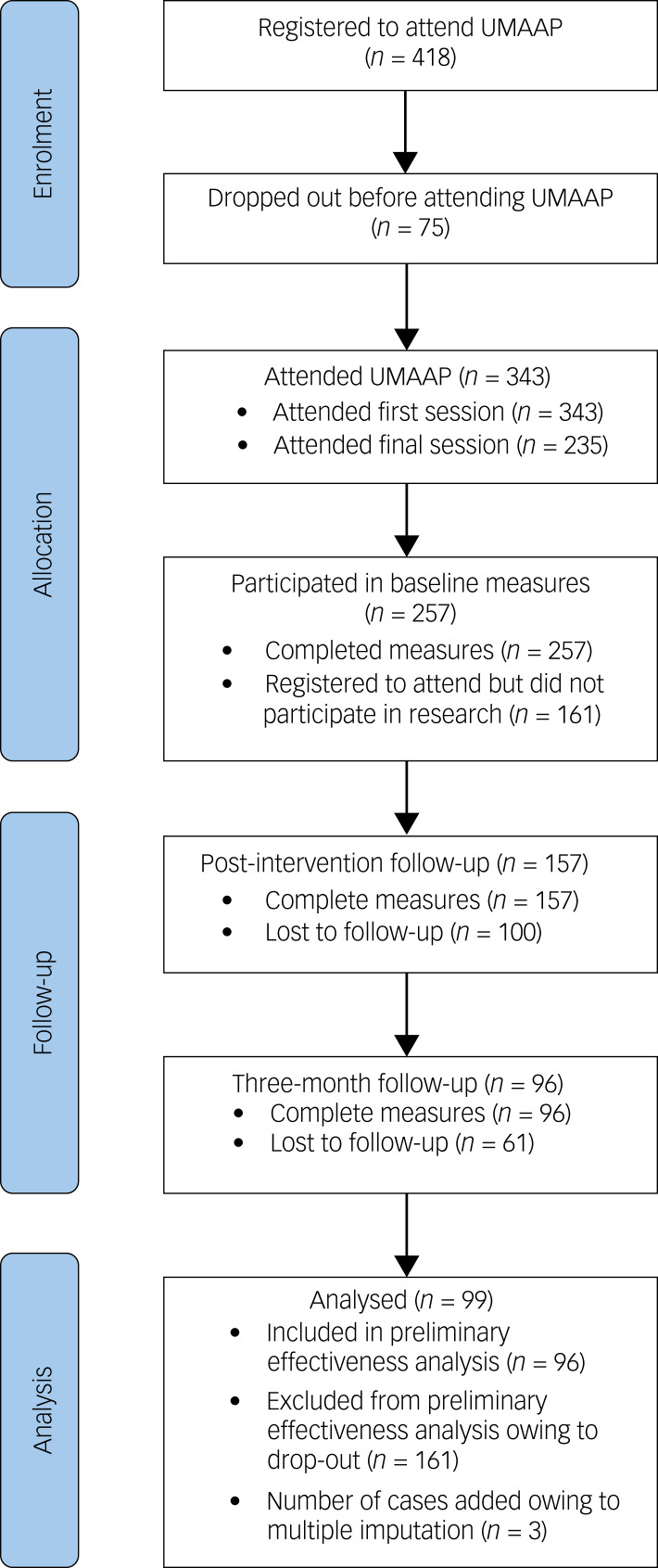
Flowchart of participation from registration to analysis. UMAAP, Understanding and Managing Adult Attention-deficit hyperactivity disorder Programme.

Of those who completed time point 1, 157 (61% retention rate) people participated in time point 2. Individuals who completed the post-UMAAP measures were invited to complete time point 3, with 96 (37% of the original participants) people participating in the final data collection. The participant attrition rate between rounds of data collection was 39%. See Fig. 1 for the participant flowchart.

### Acceptability and feasibility

**Fig. 2:**
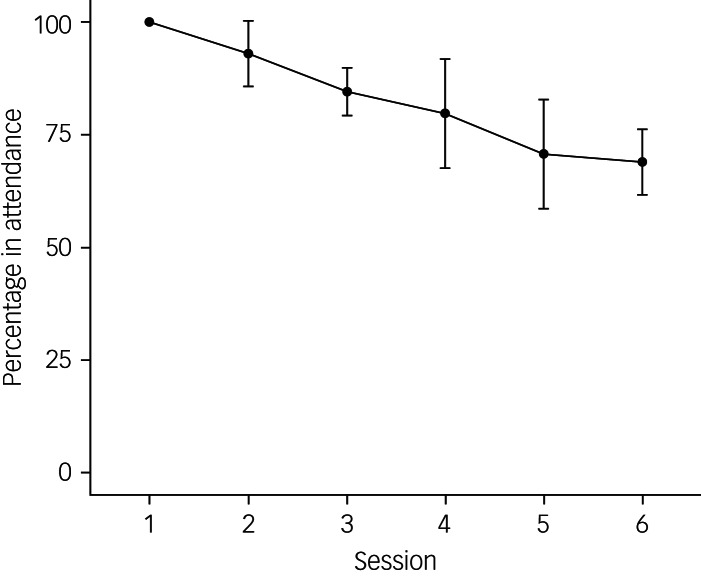
Visualisation of the average percentage of attendance in individuals who attended the first session across the programme.

Of the 418 adults with ADHD who registered to attend UMAAP, 18% (*n* = 75) of registrants dropped out of UMAAP before attending the first session. The average attendance rating across the sessions was 84% (*n* = 288) of individuals who attended the first session (*n* = 343), with a range of 69–100% and the lowest attendance in session 6 (69%, *n* = 235). Figure 2 provides a visualisation of attendance rates.

In terms of acceptability within sessions, a paired-samples *t*-test indicated that attendees rated their ADHD as being significantly less challenging at the end of each session (mean 3.93) compared with the beginning of the session (mean 4.04, *t*(53) = 4.05, *P* < 0.001, *d* = 0.20). Most attendees (91%) reported that the session helped alleviate the challenges associated with their ADHD. An exploratory one-way ANOVA suggested that some weeks had lower ratings of challenge at the end of the session (*F*(5, 48) = 2.71, *P* = 0.031). Attendees’ average end-of-session ratings of how challenging their ADHD felt remained high in the first half of UMAAP (session 1 mean 4.13, session 2 mean 4.04, session 3 mean 4.01), and appeared to reduce toward the end of the programme (session 4 mean 4.83, session 5 mean 3.87, session 6 mean 3.66). It may be that attendees found the topics in the latter half of the programme more helpful in reducing the challenges associated with their ADHD, or that they were gaining familiarity with the content and therefore finding it more helpful.

Confidence in completing the home practice was moderate (mean 3.28, s.d. = 0.26, range 2.79–4.07). The mean perceived helpfulness rating of the individual sessions was 4.23 (s.d. = 0.26), with a five being the highest rating. The average total satisfaction score was 25.37 (range 23.20–26.75), indicating an ‘excellent’ satisfaction level. As the cohort sizes gradually increased, exploratory analysis using a Pearson's correlation was conducted to investigate whether cohort size was related to perceived helpfulness. No significant relationship was observed (*r*(1,55) = −0.177, *P* = 0.195), indicating that the number of attendees did not affect satisfaction with UMAAP.

In terms of factors that may contribute to attrition, a *χ*^2^-test of independence showed that individuals diagnosed with ADHD were significantly more likely to have completed the follow-up measures than those who self-identified as having ADHD *(χ*^2^(1, 252) = 4.30, *P* = 0.038). No other variables were significantly associated with drop-out. Table 2 provides an overview of the exploration of potential differences between participants who only completed baseline measures and those who completed one or more of the follow-up measures.

**Table 2:** Independent samples *t*-tests and *χ*^2^-tests of independence investigating potential differences between participants who did and did not complete the post-intervention follow-up

Variable	Statistic^a^	Significance	Complete			Non-complete		
			*n*	Mean	s.d.	*n*	Mean	s.d.
Overall QoL	−0.18	0.86	150	34.77	10.91	98	34.52	11.01
QoL life productivity	0.63	0.53	151	28.86	17.38	99	30.38	20.35
QoL psychological health	−0.97	0.33	153	29.50	15.20	99	27.62	14.78
QoL life outlook	−0.67	0.51	152	42.49	13.60	98	41.25	15.54
QoL relationships	0.12	0.91	150	38.62	17.69	98	38.88	16.80
Self-acceptance	0.11	0.92	152	2.79	0.47	101	2.80	0.45
Psychological flexibility	0.62	0.53	145	2.74	0.76	89	2.80	0.78
Knowledge of ADHD	0.32	0.748	153	5.61	1.70	93	5.69	1.82
Age								
Gender	1.97	0.37						
Female			107			80		
Male			37			20		
Non-binary person			4			1		
ADHD status	4.30	0.038^b^						
Formally diagnosed			110			61		
Self-identified			41			40		
ADHD type	1.75	0.42						
Combined			63			37		
Inattentive			24			20		
Hyperactive–impulsive			2			3		
Additional neurodivergent condition	5.72	0.06	79			37		

QoL, quality of life; ADHD, attention-deficit hyperactivity disorder.

a. Independent samples *t*-tests (*t*) were used for continuous variables; *χ*^2^-tests of independence (*χ*^2^) were used for categorical variables.

b. *P* < 0.05.

### Preliminary effectiveness

Repeated-measures ANOVAs were used to explore the effectiveness of UMAAP on quality of life, psychological flexibility, self-acceptance and knowledge of ADHD (*n* = 99). Results indicated that scores were significantly improved after UMAAP for all outcomes (see Table 3 for results of repeated measures ANOVAs from baseline to post-intervention and final follow-up).

**Table 3:** Results of repeated-measures analyses of variance from baseline to post-intervention and final follow-up

Outcome measure	Baseline	Post-UMAAP	Three-month follow-up	rmANOVA statistics	*P*-value	*η*^2^_p_
	Mean (s.d.)	Mean (s.d.)	Mean (s.d.)	*F* (d.f.)		
Overall quality of life	34.73 (10.79)	44.13 (6.56)	45.14 (12.24)	50.73 (2, 196)	<0.001	0.34
Quality of life: life productivity subscale	29.43 (18.60)	35.05 (15.55)	36.28 (15.95)	9.77 (2, 196)	<0.001	0.09
Quality of life: psychological health subscale	28.73 (14.90)	39.65 (14.26)	42.50 (16.89)	29.33 (2, 196)	<0.001	0.23
Quality of life: life outlook subscale	41.99 (14.23)	47.83 (13.50)	50.43 (14.58)	14.62 (2, 196)	<0.001	0.13
Quality of life: relationships subscale	38.74 (17.10)	53.99 (14.10)	50.43 (14.58)	16.24 (2, 196)	<0.001	0.14
Psychological flexibility	2.79 (0.76)	2.91 (0.76)	3.20 (0.66)	8.78 (2, 190)	<0.001	0.08
Self-acceptance	2.79 (0.46)	2.99 (0.55)	3.10 (0.47)	15.56 (2, 196)	<0.001	0.14
Knowledge of ADHD	5.65 (1.74)	7.11 (1.54)	7.26 (1.74)	46.11 (2, 184)	<0.001	0.33

UMAAP, Understanding and Managing Adult ADHD Programme; rmANOVA, repeated-measures analysis of variance; ADHD, attention-deficit hyperactivity disorder.

**Fig. 3:**
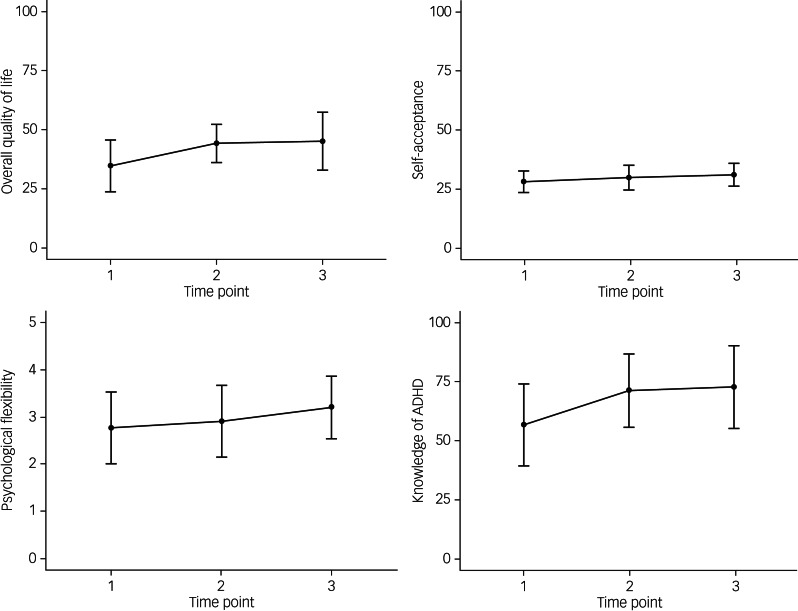
Visualisation of outcome variable scores across baseline (time point 1), post-intervention (time point 2) and three-month follow-up (time point 3). ADHD, attention-deficit hyperactivity disorder.

*Post hoc* analyses demonstrated that post-intervention scores for overall quality of life, quality-of-life subscales, knowledge of ADHD and self-acceptance were significantly higher than scores at baseline UMAAP, with effects maintained 3 months later. In the case of psychological flexibility, there was no significant difference between scores at baseline and post-intervention, but there was a significant difference between scores at the 3-month follow-up with baseline. *Post hoc* analyses are reported in Supplementary File 2. Figure 3 provides a visualisation of scores across time for overall quality of life, psychological flexibility, self-acceptance and knowledge of ADHD.

## Discussion

This pilot study aimed to investigate the feasibility of a novel psychological intervention for adults with ADHD, UMAAP. UMAAP is a community-based intervention hosted as a series of six webinars. Feasibility was evaluated in terms of suitability, such as attendance rates, engagement, understanding of the intervention, acceptability, satisfaction and preliminary effectiveness. Attendance ratings of the sessions were high, with an average of 84%. There was moderate confidence in completing the home practice. Participants rated the helpfulness of each session highly, and overall satisfaction with UMAAP was excellent. Quality of life, self-acceptance and knowledge of ADHD increased after attending UMAAP, with effects maintained at the 3-month follow-up. Although psychological flexibility did not increase immediately post-intervention, it was significantly improved 3 months later. The high attendance rate, perceived helpfulness and satisfaction with the programme, and preliminary evidence of effectiveness indicate that UMAAP is a feasible intervention for adults with ADHD.

### Feasibility

The present study demonstrated that adults with ADHD found the UMAAP sessions helpful and were satisfied with the programme overall, which was considered ‘excellent’. Participants’ satisfaction ratings were similar to other psychological interventions for adults with ADHD, such as Nasri et al^34^ and Kenter et al,^14^ indicating that UMAAP is as acceptable as an intervention for adults with ADHD as other psychological interventions. Additionally, the exploratory analysis suggested no relationship between the increased cohort sizes and the perceived helpfulness of sessions. This supports the potential of open-access, scalable psychological interventions, as satisfaction ratings were not impaired by cohort sizes of 50 people, indicating the feasibility of large online groups. However, it is worth noting that participants in the qualitative evaluation reported finding the large number of attendees and the webinar style disappointing, and would have preferred more opportunities for discussion with other attendees.^20^ Therefore, although there was no significant relationship observed, increasing the size of cohorts should be done cautiously. Interventions should consider providing the chance for participants to engage with one another in small breakout rooms to maintain a sense of connection.

Average attendance was 84% of people who attended the first session, with the lowest attendance in the final session (69%). The average attendance reflected other evaluations of psychological interventions for adults with ADHD, who have had attendance or completion ratings ranging from 65 to 85%.^8,14,34,35^ As demonstrated by Fig. 2, attendance dropped throughout the programme. Lawler and colleagues^36^ qualitatively explored individuals’ reasons for dropping out of iCBT for depression and anxiety. They observed a tendency for individuals to either feel ready to leave treatment early or have negative reasons for a change in motivation to engage. Participants who felt ready to leave iCBT early reported feeling like they had gained what they needed to from the intervention and did not need to continue. In comparison, those who dropped out for negative reasons reported not being in the right state of mind, contextual obstacles, a poor fit with iCBT, finding sessions difficult to understand, a disconnect with the content and facilitator and a lack of support.^36^ It would be valuable to explore the reasons for the 31% drop-out by the end of UMAAP with the individuals who discontinued, to explore whether changes are necessary to improve suitability.

Participants who self-identified as having ADHD were more likely to drop out than individuals who reported being diagnosed with ADHD. It could be that through attending some UMAAP sessions, participants realised that their experiences are not consistent with ADHD and therefore disengaged. Alternatively, people diagnosed with ADHD may have been receiving pharmacological treatment or additional psychological support at the time of the study, which supported their executive functioning abilities to continue with both the programme and the research. Additionally, many adults with ADHD will have experiences with adult mental health teams.^37^ Many individuals with a formal diagnosis will have been referred and reviewed by adult mental health teams, likely completing courses of other interventions such as CBT. They may be motivated to find additional support and alternative interventions, as suggested by UMAAP's qualitative evaluation.^20^ In comparison, people who self-identify are at an earlier stage of their journey and therefore may have different priorities, such as seeking an assessment or learning about ADHD more generally. Our findings could also suggest that individuals who are already diagnosed are motivated to engage in help-seeking, as they may have an increased awareness of challenges associated with their ADHD and would like to identify new ways of supporting themselves. It might be valuable to explore the feasibility of cohorts dedicated to individuals at different stages of their journey, such as a group catered to those who were diagnosed several years ago versus a group for individuals recently diagnosed or who newly self-identify. A participant in the qualitative evaluation reported that some people attending were still accepting their ADHD and were in a different phase, which limited how detailed the programme could be.^20^ Dedicated cohorts might help retention of individuals who self-identify, possibly reducing any overwhelming feelings and allowing more time to emphasise executive functioning skills, whereas cohorts for those who have been diagnosed for some time could facilitate more time to explore psychological skills. Adults with ADHD who have completed UMAAP should be consulted to provide feedback on the feasibility of dedicated versus mixed cohorts.

Although participants thought sessions were helpful, they rated their confidence in completing the home practice as moderate. The qualitative evaluation of UMAAP identified some aspects of the programme that could be overwhelming. In particular, the home practices seemed to trigger feelings of shame, and participants reported that their ADHD impaired their ability to complete the practices.^20^ Other psychological interventions have demonstrated similar challenges in incentivising participants to engage with home practices. For example, participants of a CBT for ADHD programme completed less than half of home assignments,^35^ and the majority of adults with ADHD in an emotion regulation intervention partially completed their assignments.^8^ Given that participants in the present study were only moderately confident in their ability to complete the home practice, it may be more feasible for psychological interventions such as UMAAP to exclude home assignments.

Alternatively, our findings could signal the importance of adapting home practices for neurodivergent people. The lowered confidence in completing home practices could suggest the instructions were unclear. For example, the home practice often asked attendees to identify a valued action or mindfulness practice to undertake over the week. It might be more suitable for ADHD-related executive functioning differences to provide specific instructions on the home practice. Intervention developers could also consider ‘tiered’ home practices, in which there are different options for experiential practice with increasing levels of demand. For instance, a simpler home practice might be to practice dropping anchor or mindful noticing when feeling overwhelmed by executive functioning differences, compared with a more demanding practice of daily tracking executive functioning differences and identifying what self-help tools alleviate discomfort. This way, adults with ADHD can choose a home practice they feel will be most suitable and manageable. Future research should investigate appropriate methods of adapting homework for neurodivergent people, as it is an important element of third-wave CBTs. Qualitative research with adults with ADHD would help to inform adaptations and clarify what elements of homework adults with ADHD find challenging. It would also be valuable to explore the effects of including or excluding homework in treatment dismantling or component studies.

### Preliminary effectiveness

The preliminary evaluation of effectiveness indicated that participants’ scores in quality of life, self-acceptance and knowledge of ADHD improved post-intervention, with the increase in scores maintained at 3-month follow-up. Although some research evaluations of interventions for adults with ADHD have been associated with increased quality of life post-treatment,^8,9,14^ others have not observed any significant difference.^17,35^ UMAAP participants described the therapeutic effect of psychoeducation on ADHD and ACT principles, with a particular emphasis on valued living.^20^ It may be that connecting to values, a core aspect of ACT interventions, helped to bring more meaning to participants’ lives, increasing their quality of life.

Similar to other psychoeducational interventions that showed improvements in knowledge of ADHD,^9,38^ participants rated their knowledge as significantly higher after completing UMAAP. As psychoeducation is a key first step in providing support for adults with ADHD,^3^ the increase in knowledge of ADHD suggested UMAAP may be effective as a psychoeducational resource. Therefore, clinicians can refer the individuals they work with to UMAAP to learn more about ADHD when they are newly diagnosed. Additionally, providing an open-access psychoeducational intervention could be effective in preserving time for clinicians and reducing costs, as general practitioners can save time by recommending UMAAP as a first step in place of referring individuals to adult mental health teams.

The significant increases in self-acceptance and psychological flexibility may highlight the benefits of third-wave CBTs for adult ADHD. A mixed-methods evaluation of mindfulness-based cognitive therapy for ADHD found that following the intervention, participants reported responding to their ADHD with kindness and acceptance, rather than with criticism.^39^ This is similar to the effects described by UMAAP participants, who appeared to relate to the ACT principles and neurodivergent-affirmative paradigm adopted by the intervention.^20^ The effects on self-acceptance described by UMAAP participants were supported by the present study's findings, which observed a moderate increase in self-acceptance scores.

As with the present study, adults with ADHD who participated in DBT did not have higher psychological flexibility scores immediately post-treatment, but had a significant improvement at the 3-month follow-up.^6^ It may be that there are limited effects immediately post-treatment, but as adults with ADHD practice techniques following interventions such as UMAAP, they begin to increase their psychological flexibility. Research on ACT has similarly demonstrated a nuanced incubation effect, where therapeutic effects (such as increases in psychological flexibility) appear to grow stronger with time post-intervention.^40^

Overall, the preliminary evidence of effectiveness suggests that UMAAP meets its aims of increasing quality of life, psychological flexibility, knowledge of ADHD and self-acceptance in adults with ADHD.

### Limitations and future research

The primary limitation of the study was the attrition between data collection rounds, as only 39% of individuals who participated in baseline measures completed the final round of data collection. However, there was higher retention for the intervention itself, with 69% of individuals who attended the first session attending the final session. We are unsure if the adults with ADHD who dropped out of the research are the same attendees who stopped attending UMAAP. Other intervention studies with adults with ADHD have had research retention rates ranging from 55 to 100%.^8,14,34,35^ As the research attrition rate is higher in the present study, this could highlight challenges with the research study's feasibility. The research team offered to complete the surveys by telephone or Zoom to increase flexibility and accessibility, although there was little uptake of this. The email reminders may have been unsuitable, and text reminders might have been preferred. It is possible the length of the surveys also impacted participation. Alternatively, the assistant psychologist invited anyone who registered for UMAAP to participate in the research, whereas the first author emailed the follow-up surveys. This was so we could encourage individuals who dropped out of UMAAP or who had negative experiences to continue to participate. However, participants may have missed or ignored the second or third data collection emails as a result of not recognising the first author's email. Unfortunately, the feasibility of the research methods was not measured, and therefore it is unclear why so many participants dropped out of the study. Further engagement with experts by experience or a public and patient involvement (PPI) panel could have identified challenges with the research methods and mitigation strategies. Future studies evaluating community-based or open-access interventions should ensure a measure of research feasibility and utilise PPI input to reduce attrition in efficacy-based studies. Given the drop-out between rounds, findings on the preliminary effectiveness are limited in terms of generalisability and should be interpreted cautiously.

To further ascertain the effectiveness, future research should compare UMAAP with a control group. Additionally, we did not ask participants about how many exercises they engaged in during the sessions, whether they read or completed the accompanying guidebook, or home practices outside of sessions. Further in-depth measures of engagement with the material and home practices could have provided further insight into the feasibility, and been beneficial in operationalising dosage by combining engagement with attendance rates.

Participants were asked to self-report if they had a diagnosis of ADHD or if they self-identified as having ADHD. However, we did not verify ADHD status by using a screening measure at baseline, which may have helped to support self-identification or allowed for the exclusion of individuals who do not meet the cut-off for ADHD traits. Participants were not asked questions on medication usage, current or past participation in therapy or psychological interventions, to assess whether these factors affected outcomes. Likewise, we asked participants for limited demographic details on age, gender and other neurodivergence. Additional details on employment, education and years diagnosed would have allowed us to understand who is attending, completing and benefitting the most from UMAAP. Future research is needed to understand the effects of additional factors, such as medication usage or demographic details, on the feasibility of UMAAP.

Bonferroni corrections were not applied to the *post hoc* analysis given the pilot nature of the study, although exploratory analyses in which the correction was applied were conducted and there was no difference in results. The measure of self-acceptance, DSES, had moderate internal reliability. As such, results relating to self-acceptance and the *post hoc* analyses should be interpreted with caution. Future studies could seek to develop or adapt a scale on acceptance of ADHD. Additionally, we measured knowledge of ADHD by asking participants to rate their knowledge on a scale of 1–10. In comparison, other studies have evaluated knowledge using a brief quiz.^9,38^ A limitation of our approach is relying on participants’ self-report of their knowledge, rather than measuring an objective increase. We did not create a quiz, as we were concerned that a quiz may be measuring the ability to remember specific facts, rather than learning from the psychoeducation. Future research could seek to identify and review measures of psychoeducation effectiveness in the ADHD literature to identify an appropriate common approach, as it is difficult to generalise findings across interventions.

UMAAP aims to provide a psychological intervention at the community level and, in doing so, potentially decrease the burden on secondary and tertiary services. Additional studies could employ measures of mental health difficulties that are commonly experienced by adults with ADHD, like anxiety and depression,^2^ as reductions in mental health challenges could help to reduce referrals to adult mental health teams. Going forward, the research team plans to continue to evaluate UMAAP, particularly after adapting the material and content to improve suitability. Future research projects will incorporate more demographic details to assess who is engaging with UMAAP, and the impact of medication use or experience of therapy. The team aim to provide training on UMAAP for other countries and facilitate cross-cultural research. We would like to explore the role of the facilitator's lived experience by examining outcomes in component studies comparing a therapist with ADHD versus a neurotypical therapist who co-facilitates with a peer who has ADHD. We also hope to adapt and tailor UMAAP for target groups with ADHD, such as college students or women with ADHD experiencing peri- and post-natal mental health difficulties.

In conclusion, the UMAAP is an online, group-based psychological intervention that incorporates psychoeducation with ACT principles. The intervention is facilitated by a non-profit organisation to increase access to psychological support for adults with ADHD. The present study highlighted the feasibility of attending UMAAP, and that adults with ADHD are satisfied with the intervention. Like other psychological interventions for adults with ADHD, there was moderate confidence in completing the home practices, indicating that home practices may need to be adjusted or excluded to improve feasibility. UMAAP demonstrated preliminary effectiveness in improving psychological well-being. Overall, UMAAP is feasible as an intervention. Findings suggest the benefits of offering accessible, self-referral-based psychological interventions for adults with ADHD. Further research is needed to evaluate UMAAP compared to a control group, to assess efficacy.

## Supporting information

Seery et al. supplementary material 1 Seery et al. supplementary material

Seery et al. supplementary material 2 Seery et al. supplementary material

## Data Availability

The data that support the findings of this study, analytic code/outputs and research materials are available from the corresponding author, C.S., upon reasonable request.
