# Supplementary material for: Feasibility of the Understanding and Managing Adult ADHD Programme: open-access online group psychoeducation and acceptance and commitment therapy for adults with attention-deficit hyperactivity disorder
Source: BJPsych Open. 2024 Sep 26;10(5):e163. doi: 10.1192/bjo.2024.743 (PMC11457231; doi:10.1192/bjo.2024.743)
Supplement: Seery et al. supplementary material 1 — Seery et al. supplementary material [file S2056472424007439sup001.docx]

**Intervention protocol**

***Session 1 – Foundations of UMAAP***

The first session introduces the house rules for UMAAP and the intervention’s aims of increasing understanding of adult ADHD, giving skills and techniques to thrive with ADHD, and cultivating self-compassion and awareness of what matters to the person, to help them take action towards a meaningful life with adult ADHD. The ideas behind psychoeducation and ACT are briefly introduced. Psychoeducation on the neurodiversity paradigm, ADHD as a form of neurodivergence, and the prevalence of ADHD are provided. Participants are then invited to complete an exercise exploring the attitudes about ADHD they have held and heard from others throughout their lives in their guidebook. Brown’s (2006) model of executive functioning differences is described, followed by psychoeducation on self-management styles, adapted from Merwin et al.’s (2019) metaphor of the attuned self-parent, with examples tailored to ADHD. The importance of self-care for adults with ADHD is highlighted using the acronym ‘FREES’ – Food, Rest, Exercise, Enjoyment, Socialisation. Psychoeducation on nutrition and ADHD, along with healthy techniques are provided.

**Session 2 – ADHD Acceptance**

Attendees receive a brief refresher on executive functioning differences and self-management styles. Accepting Adult ADHD is explored with reference to Kübler-Ross’s (1969) five stages of grief. The ACT ‘acceptance’ process is described, supported by the Chinese Finger Trap and letting go of the struggle metaphors. ACT’s approach to psychological health is described, with psychoeducation on the core concept of psychological flexibility. Values-guided actions, using the metaphor of towards and away moves, are highlighted as a way of creating a more meaningful life. Psychoeducation on sleep and ADHD, along with sleep hygiene and relaxation techniques are provided.

**Session 3 – Values and Vulnerabilities**

Attendees are introduced to how the messages people will receive about their differences and misunderstood ADHD traits can impact their views of themselves, based on Solden and Frank’s (2019) identity tangles. A model of three identity tangles is presented, adapted to incorporate ACT principles and processes: Tangle 1: How I respond to the unwanted experiences inside of me, Tangle 2: how I respond to my self-interpretations, and Tangle 3: how I respond with towards or away moves. Tangle 1 explores how unwanted inner experiences are normal and inevitable, and that we can create struggles for ourselves depending on how we respond to them. Fusion to thoughts, described using metaphors of becoming blinkered or stuck in unwanted thoughts, or the Pink Elephant Trap, is compared with noticing the thoughts and coming back to consciously letting values guide our actions. Tangle 2 highlights how we can misunderstand ADHD traits as personal failings or character flaws and become fused to these interpretations. Tangle 3 describes how our urges to get away or control unwanted thoughts and emotions can lead to us neglecting our values. Skills for becoming ‘untangled’ are explored, such as present-moment connection, learning about ADHD traits, and identifying values. Values are further explored, and participants are invited to complete a values card sorting exercise to identify their most important values in their guidebook. Psychoeducation on healthy exercise and ADHD are provided.

**Session 4 – Unwanted Thoughts and Self-Compassion**

Attendees receive a brief review of values, with an invitation to complete an exercise in their guidebook to identify valued living, adapted from Lundgren’s Bull’s Eye worksheet. Psychoeducation on the ACT approach to unwanted thoughts is provided, highlighting that the ultimate aim is to move from a place of thoughts dictating our actions and towards our values consciously guiding our thoughts. Attendees are invited to complete an exercise in which they reflect on an unwanted thought related to their ADHD, and what it’s like when they are blinkered by the thought, struggle against the thought, or notice the thought and return to values-guided action. Different defusion techniques, such as noticing the thought, the leaves on a stream practice, or naming the story, are provided. Psychoeducation on Neff’s (2011) approach to self-compassion is provided, along with blocks to self-compassion. Psychoeducation on hobbies and leisure and ADHD are provided.

**Session 5 – Unwanted Emotions**

Unwanted thoughts are briefly reviewed and connected to previous content on valued living. An experiential exercise is facilitated, exploring how attendees respond to their unwanted thoughts and emotions. Self-compassion is revisited and compared to self-esteem. Psychoeducation on emotion regulation and ADHD, and an ACT approach to unwanted emotions are provided. Metaphors such as the quicksand trap, under the carpet, the over-identification trap, and the deep-end trap are explained. Trich et al.’s (2011) concept of the emotional selves is explored, with references to self-management styles. Attendees are invited to complete an exercise related to the emotional selves and their responses to them in their guidebook. Techniques related to acceptance are described. Psychoeducation on hobbies and leisure and ADHD are provided.

**Session 6 – Moving Forward**

The main concepts of UMAAP are refreshed (the content related to executive functioning, identity tangles, values, accepting ADHD). Psychoeducation on socialisation and ADHD is provided, as well as mindfulness practices that are ADHD-friendly. Attendees are invited to reflect on their takeaways from UMAAP, and to complete a committed-action plan for how they will move forward after UMAAP.
