# Supplementary material for: Feasibility of the Understanding and Managing Adult ADHD Programme: open-access online group psychoeducation and acceptance and commitment therapy for adults with attention-deficit hyperactivity disorder
Source: BJPsych Open. 2024 Sep 26;10(5):e163. doi: 10.1192/bjo.2024.743 (PMC11457231; doi:10.1192/bjo.2024.743)
Supplement: Seery et al. supplementary material 2 — Seery et al. supplementary material [file S2056472424007439sup002.docx]

**Missing Data Analysis**

*Proportion of missing data by variable*

| **Variable** | **T1** | | **T2** | | **T3** | |
| --- | --- | --- | --- | --- | --- | --- |
|  | ***n*** | **%** | ***n*** | **%** | ***n*** | **%** |
| **Overall Quality of Life** | 9 | 3.5 | 102 | 39.7 | 158 | 61.5 |
| **Quality of Life: Life Productivity Subscale** | 7 | 2.7 | 100 | 38.9 | 158 | 61.5 |
| **Quality of Life: Psychological Health Subscale** | 5 | 1.9 | 100 | 38.9 | 158 | 61.5 |
| **Quality of Life: Life Outlook Subscale** | 7 | 2.7 | 102 | 39.7 | 158 | 61.5 |
| **Quality of Life: Relationships Subscale** | 7 | 2.7 | 100 | 38.9 | 158 | 61.5 |
| **Psychological Flexibility** | 23 | 8.9 | 103 | 40.1 | 158 | 61.5 |
| **Self-acceptance** | 4 | 1.6 | 100 | 38.9 | 158 | 61.5 |
| **Knowledge of ADHD** | 11 | 4.3 | 108 | 42.0 | 159 | 61.9 |

*Descriptive statistics for complete cases (CC) and multiple imputation (MI)*

| **Variable** | **T1** | | | | **T2** | | | | **T3** | | | |
| --- | --- | --- | --- | --- | --- | --- | --- | --- | --- | --- | --- | --- |
|  | **CC** | | **MI** | | **CC** | | **MI** | | **CC** | | **MI** | |
|  | ***M*** | ***SD*** | ***M*** | ***SD*** | ***M*** | ***SD*** | ***M*** | ***SD*** | ***M*** | ***SD*** | ***M*** | ***SD*** |
| **Overall Quality of Life** | 34.67 | 10.93 | 34.76 | 10.79 | 44.20 | 8.11 | 44.13 | 5.25 | 45.14 | 12.24 | - | - |
| **Quality of Life: Life Productivity Subscale** | 29.46 | 18.59 | 29.43 | 18.60 | 35.15 | 17.34 | 35.05 | 15.55 | 36.28 | 15.95 | - | - |
| **Quality of Life: Psychological Health Subscale** | 28.76 | 15.03 | 28.73 | 14.90 | 39.30 | 17.70 | 39.65 | 14.26 | 42.50 | 16.89 | - | - |
| **Quality of Life: Life Outlook Subscale** | 42.00 | 14.38 | 41.99 | 14.23 | 47.87 | 16.67 | 47.83 | 13.50 | 50.43 | 14.58 | - | - |
| **Quality of Life: Relationships Subscale** | 38.72 | 17.32 | 38.74 | 17.10 | 54.22 | 18.09 | 53.99 | 14.10 | 50.86 | 18.08 | - | - |
| **Psychological Flexibility** | 2.76 | .77 | 2.76 | .76 | 2.91 | .76 | - | - | 3.20 | .66 | - | - |
| **Self-acceptance** | 2.79 | .46 | 2.79 | .46 | 2.97 | .52 | 2.99 | .55 | 3.10 | .47 | - | - |
| **Knowledge of ADHD** | 5.64 | 1.74 | 5.65 | 1.74 | 7.11 | 1.54 | - | - | 7.26 | 1.74 | - | - |

Note = ‘-’ indicates MI was not conducted on this variable as more than 40% of data were missing

*Repeated measures ANOVA for complete cases (CC) and multiple imputation (MI)*

| **Variable** | **Repeated measures ANOVA** | | | | | | | |
| --- | --- | --- | --- | --- | --- | --- | --- | --- |
|  | **CC** | | | | **MI** | | | |
|  | ***df*** | ***F*** | ***p*** | *η^2^_p_* | ***df*** | ***F*** | ***p*** | *η^2^_p_* |
| **Overall Quality of Life** | 2, 186 | 48.09 | <.001 | .34 | 2, 196 | 50.73 | <.001 | .34 |
| **Quality of Life: Life Productivity Subscale** | 2, 188 | 10.06 | <.001 | .10 | 2, 196 | 9.77 | <.001 | .09 |
| **Quality of Life: Psychological Health Subscale** | 2, 192 | 28.50 | <.001 | .23 | 2, 196 | 29.33 | <.001 | .23 |
| **Quality of Life: Life Outlook Subscale** | 2, 190 | 14.76 | <.001 | .13 | 2, 196 | 14.62 | <.001 | .13 |
| **Quality of Life: Relationships Subscale** | 2, 192 | 15.66 | <.001 | .14 | 2, 196 | 16.24 | <.001 | .14 |
| **Psychological Flexibility** | 2, 178 | 7.18 | .003 | .08 | 2, 190 | 8.78 | <.001 | .08 |
| **Self-acceptance** | 2, 190 | 15.00 | <.001 | .14 | 2, 196 | 15.56 | <.001 | .14 |
| **Knowledge of ADHD** | 2, 182 | 44.91 | <.001 | .33 | 2, 184 | 46.11 | <.001 | .33 |

**Post-hoc pairwise comparisons for repeated measures ANOVA**

| **Outcome Measure** | **Comparison** | **Estimated Mean Difference (SD)** | **p-value** | **CI 95%** |
| --- | --- | --- | --- | --- |
| **Overall Quality of Life** | Post-intervention vs baseline | 9.69 (1.07) | <.001 | 7.57, 11.80 |
|  | Three-month follow-up vs baseline | 9.83 (1.23) | <.001 | 7.41, 12.26 |
|  | Three-month follow-up vs post-intervention | .143 (1.06) | .89 | -1.96, 2.25 |
| **Quality of Life: Life Productivity Subscale** | Post-intervention vs baseline | 7.44 (2.27) | .002 | 3.01, 11.87 |
|  | Three-month follow-up vs baseline | 7.61 (1.99) | <.001 | 3.73, 11.50 |
|  | Three-month follow-up vs post-intervention | .18 (1.70) | .89 | -3.16, 3.52 |
| **Quality of Life: Psychological Health Subscale** | Post-intervention vs baseline | 10.09 (1.74) | <.001 | 6.65, 13.54 |
|  | Three-month follow-up vs baseline | 12.50 (1.74) | <.001 | 9.05, 15.95 |
|  | Three-month follow-up vs post-intervention | 2.41 (1.72) | .16 | -1.00, 5.82 |
| **Quality of Life: Life Outlook Subscale** | Post-intervention vs baseline | 6.12 (1.54) | <.001 | 3.10, 9.14 |
|  | Three-month follow-up vs baseline | 7.23 (1.38) | <.001 | 4.49, 9.97 |
|  | Three-month follow-up vs post-intervention | 1.11 (1.44) | .44 | -1.70, 3.92 |
| **Quality of Life: Relationships Subscale** | Post-intervention vs baseline | 15.12 (3.14) | <.001 | 8.90, 21.35 |
|  | Three-month follow-up vs baseline | 12.01 (1.96) | <.001 | 4.49, 9.97 |
|  | Three-month follow-up vs post-intervention | -3.12 (3.15) | .32 | -9.57, 3.13 |
| **Psychological Flexibility** | Post-intervention vs baseline | -.01 (.13) | .94 | -.27, .26 |
|  | Three-month follow-up vs baseline | .42 (.08) | <.001 | .26, .58 |
|  | Three-month follow-up vs post-intervention | .43 (.13) | .001 | .17, .68 |
| **Self-acceptance** | Post-intervention vs baseline | .19 (.05) | <.001 | .10, .28 |
|  | Three-month follow-up vs baseline | .25 (.05) | <.001 | .15, .35 |
|  | Three-month follow-up vs post-intervention | .06 (.05) | .16 | -.02, .15 |
| **Knowledge of ADHD** | Post-intervention vs baseline | 1.55 (.16) | <.001 | 1.23, 1.82 |
|  | Three-month follow-up vs baseline | 1.42 (.19) | <.001 | 1.05, 1.80 |
|  | Three-month follow-up vs post-intervention | -.13 (.19) | .49 | -.50, .24 |
